# COX-2/sEH Dual Inhibitor PTUPB Alleviates CCl_*4*_-Induced Liver Fibrosis and Portal Hypertension

**DOI:** 10.3389/fmed.2021.761517

**Published:** 2021-12-23

**Authors:** Zhifeng Zhao, Chihao Zhang, Jiayun Lin, Lei Zheng, Hongjie Li, Xiaoliang Qi, Haizhong Huo, Xiaolou Lou, Bruce D. Hammock, Sung Hee Hwang, Yongyang Bao, Meng Luo

**Affiliations:** ^1^Department of General Surgery, Shanghai Ninth People's Hospital, Shanghai Jiao Tong University School of Medicine, Shanghai, China; ^2^Department of Entomology, Nematology and UC Davis Comprehensive Cancer Center, Davis, CA, United States; ^3^Department of Pathology, Shanghai Ninth People's Hospital, Shanghai Jiao Tong University School of Medicine, Shanghai, China

**Keywords:** PTUPB, liver fibrosis, portal hypertension, inflammation, angiogenesis

## Abstract

**Background:** 4-(5-phenyl-3-{3-[3-(4-trifluoromethylphenyl)-ureido]-propyl}-pyrazol-1-yl) -benzenesulfonamide (PTUPB), a dual cyclooxygenase-2 (COX-2)/soluble epoxide hydrolase (sEH) inhibitor, was found to alleviate renal, pulmonary fibrosis and liver injury. However, few is known about the effect of PTUPB on liver cirrhosis. In this study, we aimed to explore the role of PTUPB in liver cirrhosis and portal hypertension (PHT).

**Method:** Rat liver cirrhosis model was established *via* subcutaneous injection of carbon tetrachloride (CCl_4_) for 16 weeks. The experimental group received oral administration of PTUPB (10 mg/kg) for 4 weeks. We subsequently analyzed portal pressure (PP), liver fibrosis, inflammation, angiogenesis, and intra- or extrahepatic vascular remodeling. Additionally, network pharmacology was used to investigate the possible mechanisms of PTUPB in live fibrosis.

**Results:** CCl_4_ exposure induced liver fibrosis, inflammation, angiogenesis, vascular remodeling and PHT, and PTUPB alleviated these changes. PTUPB decreased PP from 17.50 ± 4.65 to 6.37 ± 1.40 mmHg, reduced collagen deposition and profibrotic factor. PTUPB alleviated the inflammation and bile duct proliferation, as indicated by decrease in serum interleukin-6 (IL-6), liver cytokeratin 19 (CK-19), transaminase, and macrophage infiltration. PTUPB also restored vessel wall thickness of superior mesenteric arteries (SMA) and inhibited intra- or extrahepatic angiogenesis and vascular remodeling *via* vascular endothelial growth factor (VEGF), von Willebrand factor (vWF), etc. Moreover, PTUPB induced sinusoidal vasodilation by upregulating endothelial nitric oxide synthase (eNOS) and GTP-cyclohydrolase 1 (GCH1). In enrichment analysis, PTUPB engaged in multiple biological functions related to cirrhosis, including blood pressure, tissue remodeling, immunological inflammation, macrophage activation, and fibroblast proliferation. Additionally, PTUPB suppressed hepatic expression of sEH, COX-2, and transforming growth factor-β (TGF-β).

**Conclusion:** 4-(5-phenyl-3-{3-[3-(4-trifluoromethylphenyl)-ureido]-propyl}-pyrazol-1-yl)- benzenesulfonamide ameliorated liver fibrosis and PHT by inhibiting fibrotic deposition, inflammation, angiogenesis, sinusoidal, and SMA remodeling. The molecular mechanism may be mediated *via* the downregulation of the sEH/COX-2/TGF-β.

## Background

As a prevalent and challenging illness, liver cirrhosis is characterized by abnormal buildup of hepatic extracellular matrix (ECM) as a result of inflammation or damage (1). The deposition of fibrotic tissue increases intrahepatic circulatory resistance and extrahepatic circulatory pressure which lead to portal hypertension (PHT) (2). PHT can result in esophageal and gastric varices, and also severe bleeding in the upper gastrointestinal system; thus, early management is critical. However, there is currently no specific treatment for liver cirrhosis, especially PHT.

As one of the most abundant lipid mediators, arachidonic acid (ARA) and its metabolites play a critical role in the vasoactivity, inflammation, fibrosis, etc. The ARA can be metabolized and transformed *via* three pathways: cyclooxygenase (COX), cytochrome P450 (CYP450), and lipoxygenase (LOX). Among them, COX and CYP450 pathways are most strongly associated with liver cirrhosis (2). COX is divided into two categories: COX-1 and COX-2. As a biomarker of inflammation, immune system, and cell proliferation, COX-2 was deeply involved in the progression and deterioration of liver cirrhosis (3). The CYP450 pathway performed a variety of activities, including antiinflammatory, antihypertension, and antifibrosis, mostly *via* the epoxyeicosatrienoic acids (EETs) (4–6). However, EETs are often catalyzed by soluble epoxide hydrolase (sEH) into the less biologically active metabolite in stress situations such as hypertension (7, 8). As a result, the biological activities of ARA pathways depend primarily on COX-2 and sEH.

In recent years, a COX-2/sEH dual inhibitor, PTUPB, has been developed. PTUPB was later discovered to have impacts on pulmonary fibrosis (9), renal fibrosis (10), and liver injury (11). Those studies above suggested that PTUPB shows an alleviating effect in fibrosis. But the effect of PTUPB in liver cirrhosis and PHT has not been researched yet. In this article, the effect of PTUPB on liver cirrhosis and PHT was explored.

This study is made up of the following sections. To begin, we investigated PTUPB's remission impact on hepatic fibrosis. Following that, we investigated the impact of PTUPB on liver inflammation and function. Then, we investigated the angiogenesis pathways in the liver and the influence of PTUPB on mesenteric vascular remodeling. Finally, we investigated the mechanism and pathways.

## Method

### Animals and Reagents

All animal-related protocols were approved by the Ethical Committee of Shanghai Ninth People's Hospital, Shanghai Jiao Tong University School of Medicine (Shanghai, China). Sprague Dawley (SD) rats (male, 6–8 weeks old) weighing 200–250 g were purchased from the Experimental Animal Center of School of Medicine, Shanghai Jiao Tong University (Shanghai, China). The rats were maintained in our specific pathogen-free facility under controlled conditions (22°C, 40–60% humidity, and 12-h light/dark cycle), and free access to tap water and standard rat food was given to the rats.

1-(4-trifluoromethoxyphenyl)-3-(1-propionylpiperidin-4-yl)-urea, TPPU, and 4-(5-phenyl-3-{3-[3-(4-trifluoromethylphenyl)-ureido]-propyl}-pyrazol-1-yl)-benzenesulfonamide, PTUPB, were synthesized according to the previous procedures (12). TPPU was generously provided by the laboratory of Dr. Bruce Hammock (UC Davis, USA) and stored at 20°C. TPPU and PTUPB were dissolved in PEG-400 to give a 10g/L clear solution. This solution was then added to warm drinking water with rapid stirring to give the 100 mg/L solution of TPPU/PTUPB in drinking water. Based on the estimation of daily water consumption, a concentration of 100 mg/L inhibitor in drinking water will result in a dose of approximately 10 mg/kg/day. The other rats received vehicle (PEG 400 diluted in water) as control. PTUPB and TPPU were administered for 4 weeks from the 12th week in the CCl_4_ model group.

**Figure 1:**
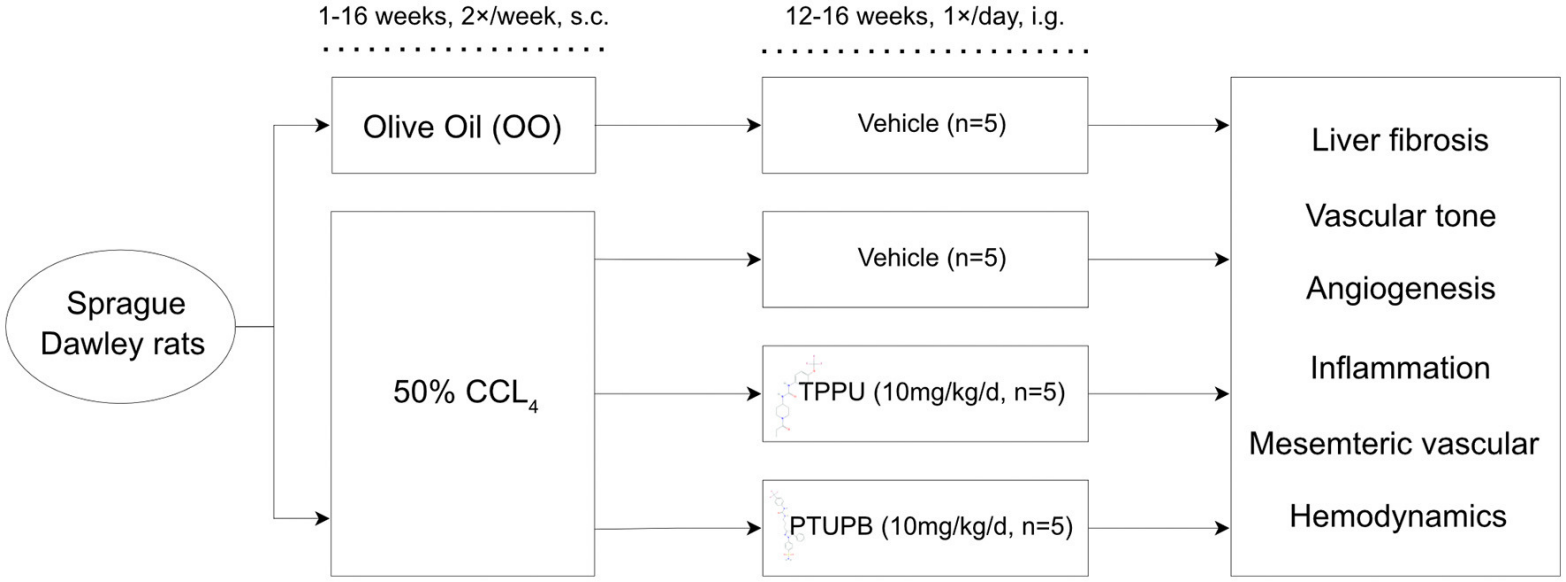
Schematic diagram of animal models.

A total of 20 rats were used in this study. Rats were divided into four subgroups as follows: control group (OO-VEH) received pure olive oil injection with vehicle administration (*n* = 5); PHT group received carbon tetrachloride (CCl_4_) (50% in olive oil, v/v, 1 ml/kg) by subcutaneous injection (s.c.) two times a week for 16 weeks with vehicle administration (CCl_4_-VEH) (*n* = 5); PHT group with TPPU administration (CCl_4_-TPPU) (*n* = 5); and PHT group with PTUPB administration (CCl_4_-PTUPB). The detailed grouping strategy was shown in Figure 1.

### Hemodynamic Measurements

After finishing modeling, rats were anesthetized with 40 mg/kg zolazepam and tiletamine (Zoletil 50, France) and 8 μg/kg dexmedetomidine hydrochloride (Dexdomitor^®^, Pfizer Inc. USA) through intramuscular injections. A PE-50 catheter (Smiths Medical, UK) was inserted into the right femoral artery to determine the heart rate (HR) and mean arterial pressure (MAP). The catheter was then placed into the portal vein to determine the portal pressure (PP). A transducer linked the catheter to the monitor, and the values were acquired using a multichannel physiological signal collection system (ALC-MPA multichannel bioinformatics analysis system, Shanghai Alcott Biotechnology Co., Ltd., China). Following sacrifice of the rats, blood, liver, and superior mesenteric arteries (SMA) were taken for enzymatic analysis, histological, and molecular analysis.

### Enzyme-Linked Immunosorbent Assay (ELISA)

Serum levels of interleukin (IL)-6 and hyaluronan were determined by IL-6 rat ELISA kit (Thermo Fisher, USA) and hyaluronan rat ELISA Kit (R&D Systems, USA), respectively, according to the manufacturer's instructions.

### Histological and Immunohistochemical (IHC) Examination

The liver sections from the right lobe and mesenteric tissues were fixed in 10% formalin buffer (pH 7.4) and embedded in a paraffin block. Hematoxylin–eosin (H&E), Masson, and Sirius Red staining were used on the liver sections, followed by random evaluation under a light microscope by an expert pathologist.

For IHC staining, liver sections were incubated with antimatrix metalloproteinase-2 (MMP-2) antibody (1:150, Servicebio, China), antimatrix metalloproteinase-9 (MMP-9) antibody (1:300, Servicebio, China), antivascular endothelial growth factor (VEGF) A antibody (1:250, Servicebio, China), antivon Willebrand factor (vWF) antibody (1:1,000, Servicebio, China), anticytokeratin 19 (CK-19) antibody (1:1,000, Servicebio, China), anti-sEH antibody (1:50, Absin, China), anti-CD31 antibody (1:300, Servicebio, China), and anti-CD68 antibody (1:150, Servicebio, China), overnight at 4°C with phosphate-buffered saline (PBS) as negative control. Subsequently, the sections were incubated with appropriate HRP-conjugated goat antirabbit secondary antibody for 60 min, followed by restaining with hematoxylin. The collagen deposition volume and stained area were calculated with IHC Profiler plugin in ImageJ (version 1.53, USA). The results were expressed as the proportions of the stained areas. The total area and average values were taken from five rats in each group.

### Hepatic Functions

Hepatic functions, including the total bilirubin (TBIL), direct bilirubin (DBIL), aspartate aminotransferase (AST), alanine transaminase (ALT), and γ-glutamyl transferase (GGT), were examined using kits from Changchun Huili Biotech (China) according to the manufacturer's instruction.

### Western Blotting Analysis

Samples from liver tissue were taken and kept at −80°C. To extract protein from the liver, the sample was crushed in liquid nitrogen and homogenized according to the manufacturer's instructions using RIPA buffer (Beyotime, China). The liver extracts were then centrifuged for 15 min at 10,000 g for 15 min at 4°C. The supernatant was immediately collected, and the BCA protein analysis kit was used to determine the total protein content (Beyotime, China). Equal amounts of proteins were electrophoresed on sodium dodecyl sulfate-polyacrylamide gels (SDS-PAGE) and then electrotransferred onto polyvinylidene difluoride (PVDF) membranes. Primary antibodies against α-smooth muscle actin (α-SMA) (1:1,000, Servicebio, China), COX-2 (1: 750, Servicebio, China), and sEH (1:1,000, Absin, China) were used to incubate the blots (1:1,000, Servicebio, China). The membranes were then treated with corresponding secondary antibodies. Immunoreactive bands were visualized using an electrochemiluminescence instrument (Vilber Lourmat, France) and quantified using digital image software (Kodak, USA).

### Real-Time Polymerase Chain Reaction (qRT-PCR)

The mRNA expressions of TGF-β, epoxide hydrolase 2 (EPHX2), COX2, α-SMA, collagen type I alpha 1 chain (COL1A1), MMP2, MMP9, VEGF, VWF, angiopoietin 1 (ANGPT1), endothelial nitric oxide synthase (eNOS), GTP cyclohydrolase 1 (GCH1), and adhesion G protein-coupled receptor E1 (Adgre1, F4-80) were determined. qRT-PCR was conducted on a Bio-Rad iCycleriQ real-time PCR detection system (Bio-Rad laboratories, Germany) using IQ SYBR Green supermix Kit (Bio-Rad). The reaction was carried out in triplicate. The data were analyzed using the iCycleriQ software system (Bio-Rad, Germany).

### Bioinformatic Analysis

Potential target genes were obtained from Pharm Mapper (http://lilabecust.cn/pharmmapper/) and SwissTargetPrediction [https://swisstargetprediction.ch/; probability value ≥0.10 was selected (13)]. Afterward, we entered the keyword “liver fibrosis” into the GeneCards (https://www.genecards.org/) (14) to obtain target genes related to liver fibrosis. The differentially expressed genes related to PTUPB and liver fibrosis were intersected and depicted in Venn diagram by *ggVennDiagram* package.

Protein–protein interaction (PPI) network of common target genes was generated from STRING database [https://string-db.org/ (15)]. The PPI network was visualized using Cytoscape (version 3.6.1) (16). To determine the hub genes, we computed the centrality of each mRNA node using “MCC” method in CytoHubba, a Cytoscape plugin (17). The top ten genes were deemed hub mRNAs based on their degree of centrality.

The *clusterProfiler* package was used to perform gene ontology (GO) and Kyoto Encyclopedia of Genes and Genomes (KEGG) pathway analysis on common target genes (18). The GO terms describe gene functions in three aspects: biological processes, molecular functions, and cellular components. The KEGG analysis predicts the involvement of the common target genes in various biological pathways. The modified *p* < 0.05 was used as a cutoff value (19, 20).

### Statistical Analysis

All the statistical analyses were performed using R (version 4.1.0.). Continuous variables were expressed as means ± standard deviation (SD). N represents the number of rats. Statistical significance was calculated by Student's *t*-test or Mann–Whitney *U* test. Two-sided *p* < 0.05 was considered statistically significant.

## Result

### PTUPB Reduces Portal Hypertension

The hemodynamic and general characteristics were measured including weight, liver weight, HR, MAP, and PP. After the modeling of PHT, CCl4-VEH group presented with a higher PP (17.50 ± 4.65 vs. 5.40 ± 1.13 mmHg), lower weight (465.75 ± 11.15 vs. 562.25 ± 65.38 g), and lower liver weights (21.63 ± 0.93 vs. 26.93 ± 1.77 g) compared with OO-VEH significantly (*p* < 0.05), although MAP and HR remained comparable. Following the treatment of PTUPB, the body and liver weights of PHT rats rebounded and PP decreased significantly from 17.50 ± 4.65 to 6.37 ± 1.40 mmHg (*p* < 0.001), whereas map and HR remained unchanged. Similar to PTUPB, TPPU treatment also increased weights (609.50 ± 25.12 vs. 465.75 ± 11.15 g) and liver weights (29.33 ± 2.76 vs. 21.63 ± 0.93 g) compared with CCl_4_-VEH group and decreased PP (6.86 ± 1.44 vs. 17.50 ± 4.65 mmHg) significantly (*p* < 0.05). The results are shown in Table 1.

**Table 1:** Hemodynamic and general characteristics.

	**OO-VEH(*n* = 5)**	**CCl_4_ -VEH(*n* = 5)**	**CCl_4_-TPPU(*n* = 5)**	**CCl_4_-PTUPB(*n* = 5)**	**OO *vs*. CCl_4_**	**CCl_4_-VEH *vs*. TPPU**	**CCl_4_-VEH *vs*. PTUPB**
Weight/g	562.25 ± 65.38	465.75 ± 11.15	609.50 ± 25.12	555.50 ± 15.59	0.013^*^	0.001^*^	0.02^*^
Liver/g	26.93 ± 1.77	21.63 ± 0.93	29.33 ± 2.76	29.93 ± 2.71	0.022^*^	0.002^*^	<0.001^*^
MAP/mmHg	82.75 ± 8.42	67.67 ± 11.59	60.75 ± 3.50	70.00 ± 4.69	0.082	0.615	0.974
PP/mmHg	5.40 ± 1.13	17.50 ± 4.65	6.86 ± 1.44	6.37 ± 1.40	<0.001^*^	<0.001^*^	<0.001^*^
HR/bpm	370.50 ± 32.69	375.75 ± 21.62	352.50 ± 60.08	388.5 ± 19.96	0.997	0.814	0.961

* *Statistical significance (p < 0.05) by Student's t-test*.

### Antifibrotic Effects of PTUPB

**Figure 2:**
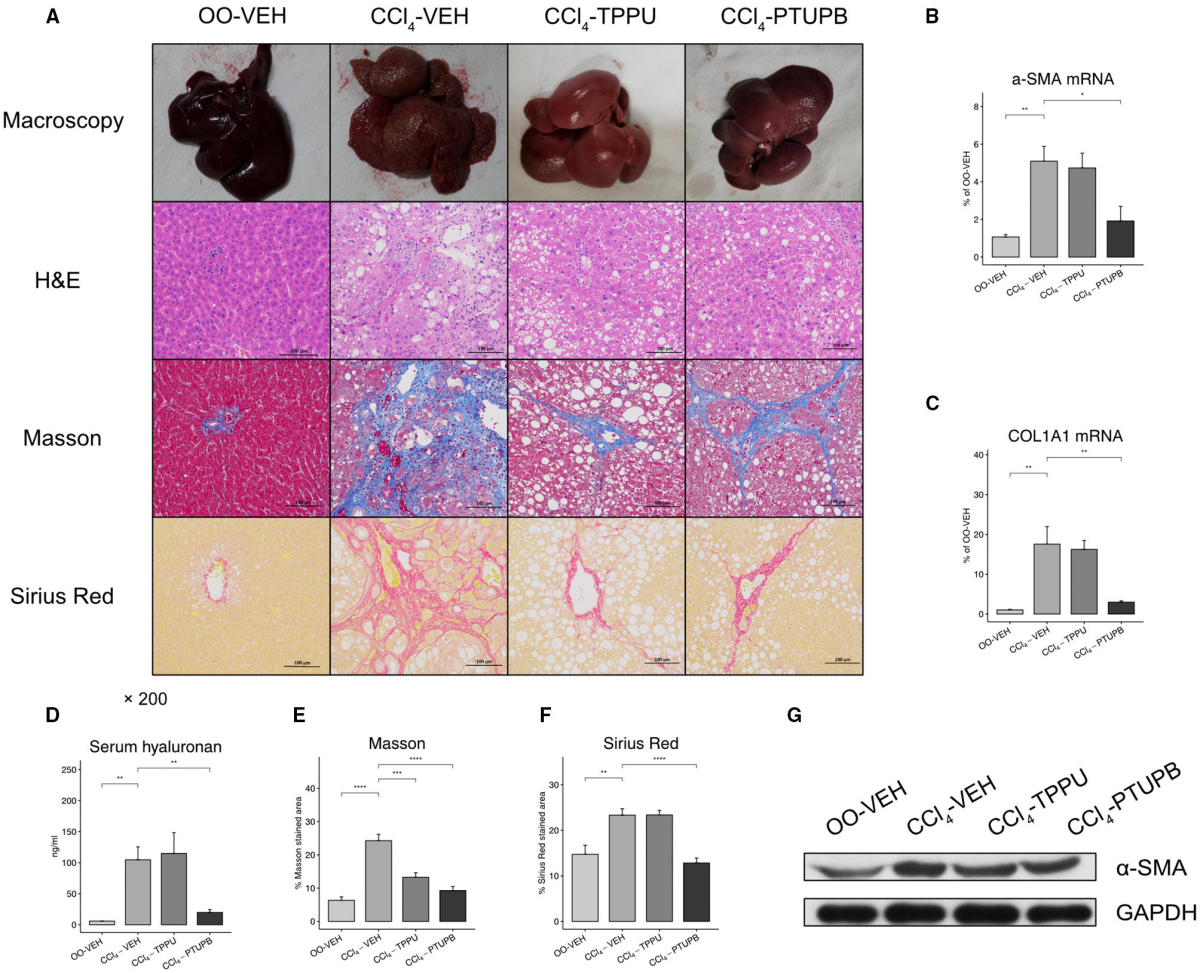
Antifibrotic effects of PTUPB and TPPU. **(A)** Macroscopic view of specimen, H&E, Masson, and Sirius Red staining. Gene expression of α-SMA **(B)** and COL1A1 **(C)** decreased in PTUPB treatment group. **(D)** Serum concentration of hyaluronan increased in CCl_4_-VEH group and downregulated in CCl_4_-PTUPB group. Quantitative analysis of Masson **(E)** and Sirius Red **(F)** showed the significant decrease in fibrotic area after the treatment of PTUPB. **(G)** Western blots showed the decrease of α-SMA in CCl_4_-PTUPB group. ((*p <0.05, **p <0.01, ***p <0.001, ****p <0.0001 vs. CCl_4_-VEH using Student's *t*-test, data are represented as mean with SD).

After 16 weeks of CCl4 treatment, rats in the CCl4-VEH group exhibited marked liver fibrosis. Liver in PHT rats showed increased vacuolar degeneration and lysis in H&E staining, more collagen deposition in Masson (24% vs. 6%) and Sirius Red (23% vs. 15%) staining (Figures 2A,E,F). The hepatic expression of α-SMA, COL1A1, and serum hyaluronan also increased significantly (Figures 2B–D). PTUPB treatment resulted in significant reduced collagen deposition in Masson (9% vs. 24%) and Sirius Red (13% vs. 23%) staining which could also been confirmed by macroscopy and H&E (Figures 2A,E,F). In addition, the hepatic expression of α-SMA, COL1A1, and serum hyaluronan also decreased significantly after PTUPB treatment (Figure 2B–**D,G**). The treatment of TPPU was relatively less effective. Only the Masson staining of CCl4-TPPU group (13%) was significantly less than CCl4-VEH. No significant difference was observed in other indicators between CCl_4_-TPPU with CCl_4_-VEH group including the Sirius Red staining, serum hyaluronan, and hepatic expression of α-SMA and COL1A1 (Figures 2B–F).

### PTUPB Ameliorate Inflammation and Liver Functions

**Figure 3:**
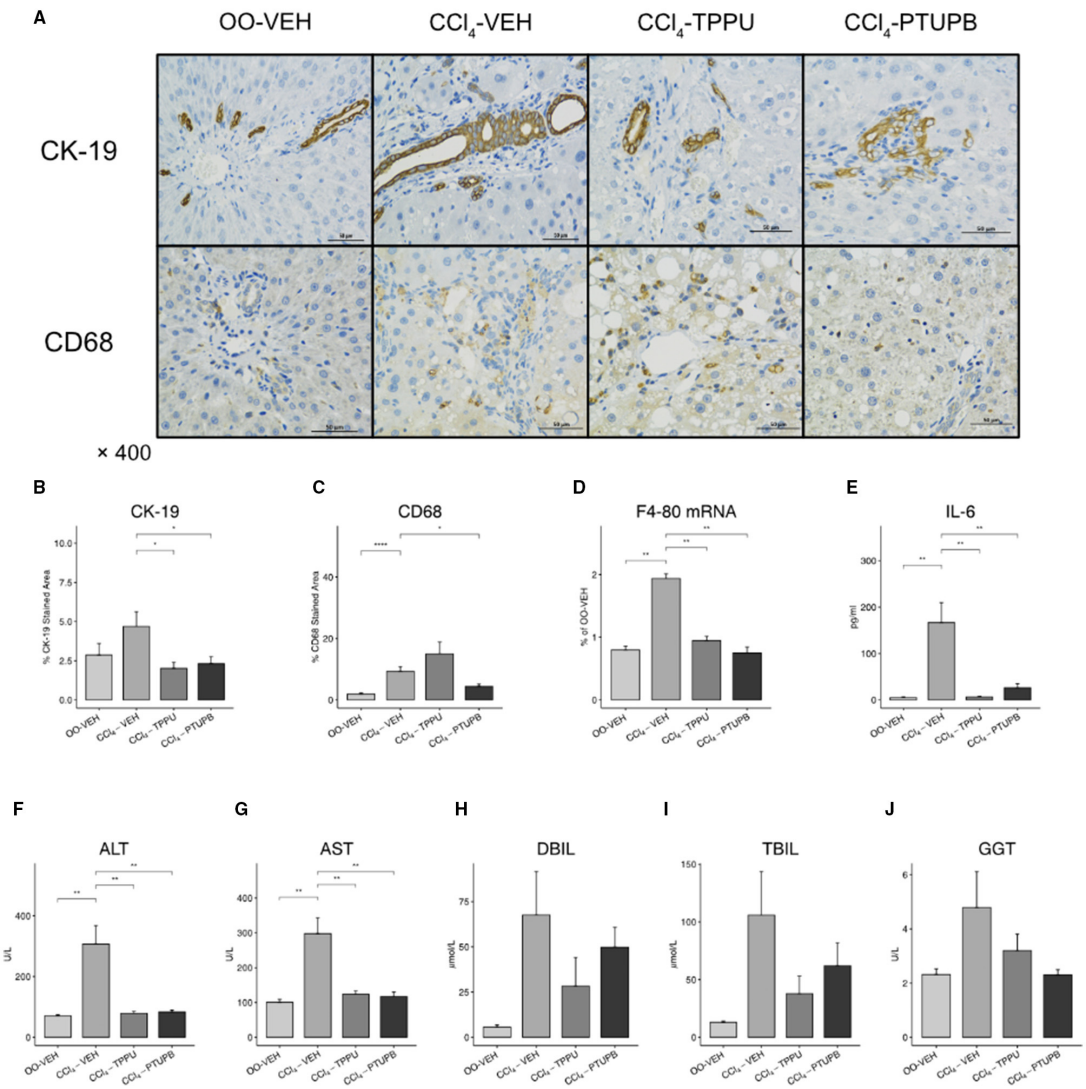
PTUPB inhibits hepatic inflammation and bile duct cell proliferation. **(A)** Immunohistochemistry (IHC) on liver tissue with anti-CK-19 and anti-CD68 antibody. Quantification of IHC staining analyzed by IHC Profiler indicated significant decrease of CK-19 **(B)** and CD68 **(C)** in liver tissue. Furthermore, levels of hepatic expression of F4-80 **(D)**, serum IL-6 **(E)**, ALT **(F)**, AST**(G)**, DBIL **(H)**, TBIL **(I)**, and GGT **(J)** were measured. F4-80, IL-6, ALT, and AST were decreased significantly after PTUPB treatment. (*p <0.05, **p <0.01, ****p <0.0001 vs. CCl_4_-VEH using Student's *t*-test, data are represented as mean with SD).

Liver fibrosis is often accompanied by an increase hepatic and systemic inflammation. In CCl4-VEH group, hepatic expression of F4/80 mRNA and CD68 staining area increased significantly, indicating the upregulated hepatic mononuclear or macrophage infiltration (Figures 3C,D). The serum IL-6 also elevated, suggesting the systemic inflammation in PHT models (Figure 3E). The PTUPB treatment groups showed significant lower levels of F4/80 mRNA and CD68 staining in liver and also serum IL-6 (Figures 3A–E). Similar but less-effective results were observed in CCl_4_-TPPU group.

In addition, we found that the liver structure was destroyed and its functions were decreased severely in PHT models. CK-19, a classical marker of bile duct cell proliferation, was upregulated in CCl4-VEH group compared with OO-VEH group (5% vs. 2%). Liver function markers, such as ALT, AST and DBIL, were also elevated significantly in CCl4-VEH rats (Figures 3A,B,F–H). In TPPU and PTUPB treatment groups, CK-19, ALT, and AST were observed to decrease significantly (Figures 3B,F,G) whereas TBIL, DBIL, and GGT did not change significantly (Figures 3H–J).

### PTUPB Inhibits Pathological Angiogenesis and Sinusoidal Remodeling

**Figure 4:**
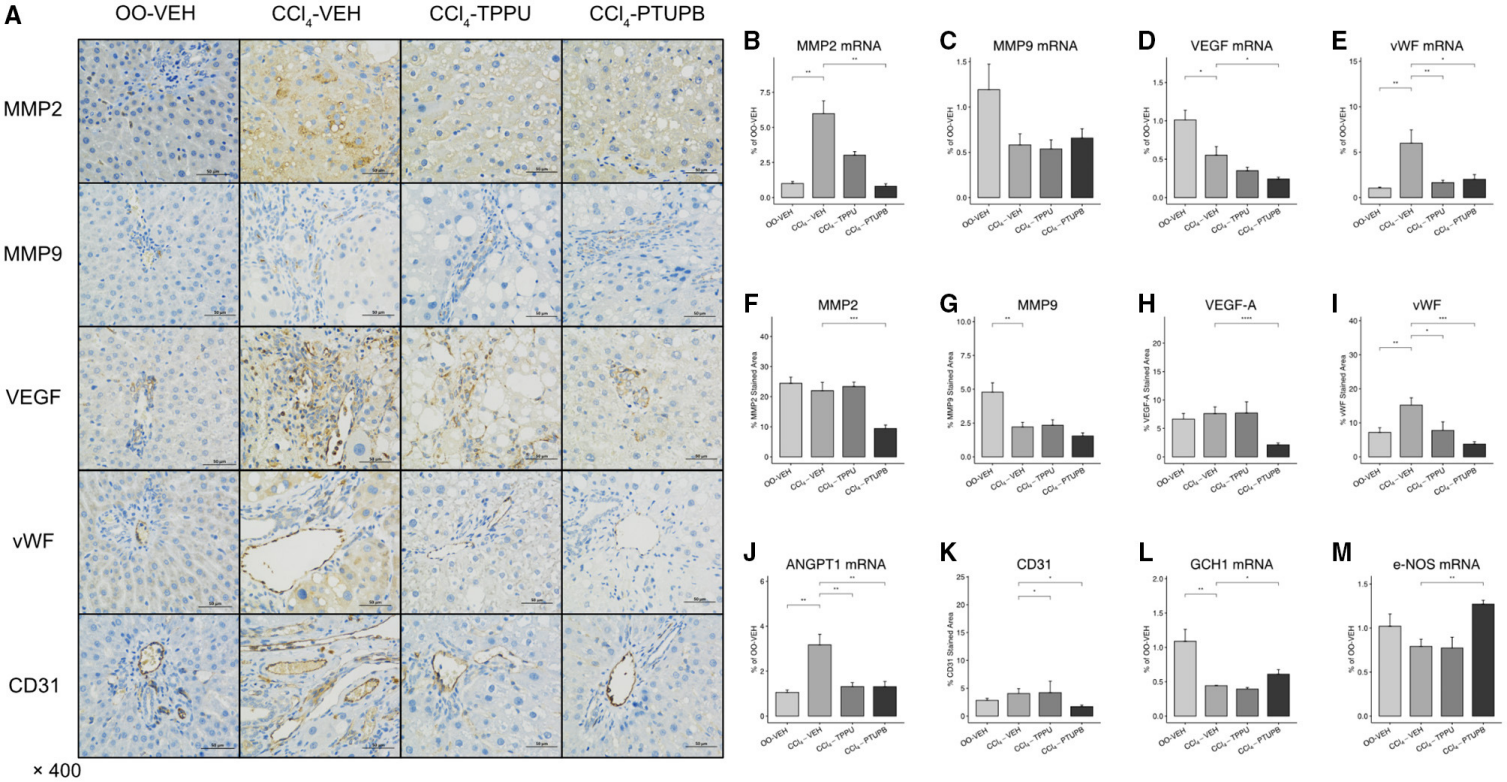
PTUPB inhibits angiogenesis, sinusoidal remodeling, and vascular tone. **(A)** IHC staining for MMP-2, MMP-9, VEGF, vWF, and CD31 in liver tissue. After the PTUPB treatment, several gene expressions decreased including MMP2 **(B)**, VEGF **(D)**, vWF **(E)**, Angpt1 **(J)**, with two gene expressions increased consisted of GCH1**(L)** and eNOS **(M)**, whereas MMP9 **(C)** showed no significant difference. Quantification of IHC staining of MMP2 **(F)**, VEGF **(H)**, vWF **(I)**, and CD31 **(K)** also decreased in CCl_4_-PTUPB group whereas MMP9 **(G)** showed no significant difference either. (*p <0.05, **p <0.01, ***p <0.001, ****p <0.0001 vs. CCl_4_-VEH using Student's *t*-test, data are represented as mean with SD).

Pathological angiogenesis and sinusoidal remodeling are remarkable pathological symptoms of cirrhosis which contribute to vascular resistance and PHT. Several mediators of angiogenesis and remodeling were significantly increased in PHT rats including MMP2, VEGF, vWF, and Angpt1 (Figures 4B–E,J), which were confirmed by immunohistochemical staining (Figure 4A). After PTUPB treatment, MMP2, VEGF, vWF, Angpt1, and CD31 were significantly reduced, whereas MMP9 remained unchanged (Figures 4B–E,J,K). However, in CCl_4_-TPPU group, only vWF, Angpt1, and CD31 were reduced significantly. In addition to the sinusoid remodeling, sinusoidal dysfunction also contributes to vascular resistance. Our results indicated that the expression of GCH1 decreased significantly in PHT group. After PTUPB treatment, the hepatic GCH1 and eNOS mRNA increased remarkably (Figures 4L,M).

### PTUPB Improves Vascular Remodeling in Mesenteric Artery

**Figure 5:**
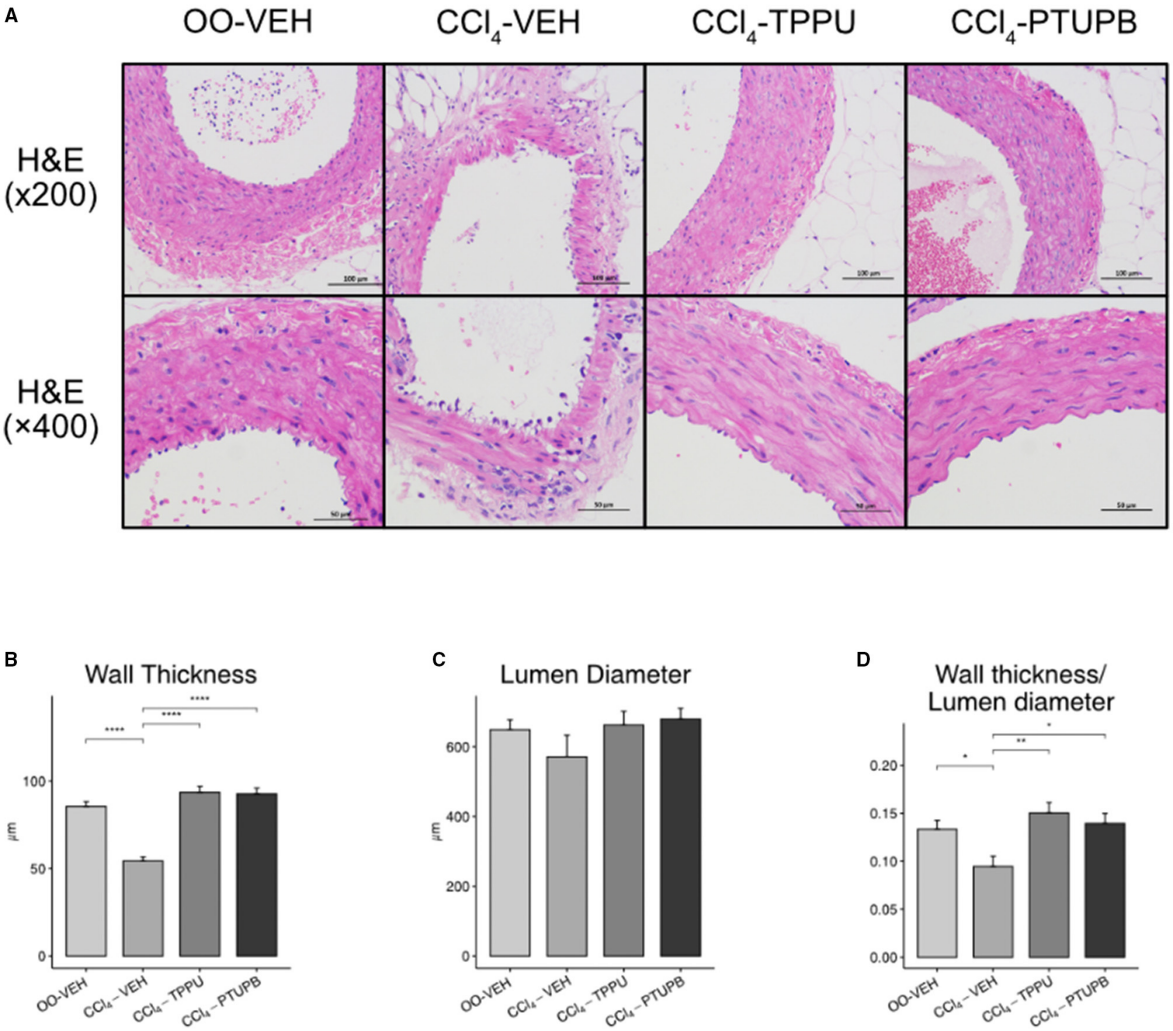
PTUPB ameliorates the vascular remodeling in SMA. **(A)** H&E staining of SMA with magnification of × 200 and × 400. **(B)** The vessel wall thickness decreased in CCl_4_-VEH rats and restored significantly in CCl_4_-PTUPB group. **(C)** No significant difference was detected among the lumen diameters of SMA. **(D)** Similar to wall thickness, the ratio of wall thickness or lumen diameter deceased in CCl_4_-VEH rats and restored significantly in CCl_4_-PTUPB group. (*p <0.05, **p <0.01, ****p <0.0001 vs. CCl_4_-VEH using Student's *t*-test, data are represented as mean with SD).

In CCl_4_-induced PHT rats, the lumen wall of SMA became thinner while its vascular pattern became disrupted (Figures 5A,B). However, the lumen diameter stayed unchanged in CCl_4_-VEH group (Figure 5C) despite the wall thickness or lumen diameter ratio still reduced remarkably (Figure 5D). Following the treatment of PTUPB and TPPU, SMA restored to normal state with thicker wall thickness and higher wall thickness or lumen diameter ratio (Figures 5A,B,D).

**Figure 6:**
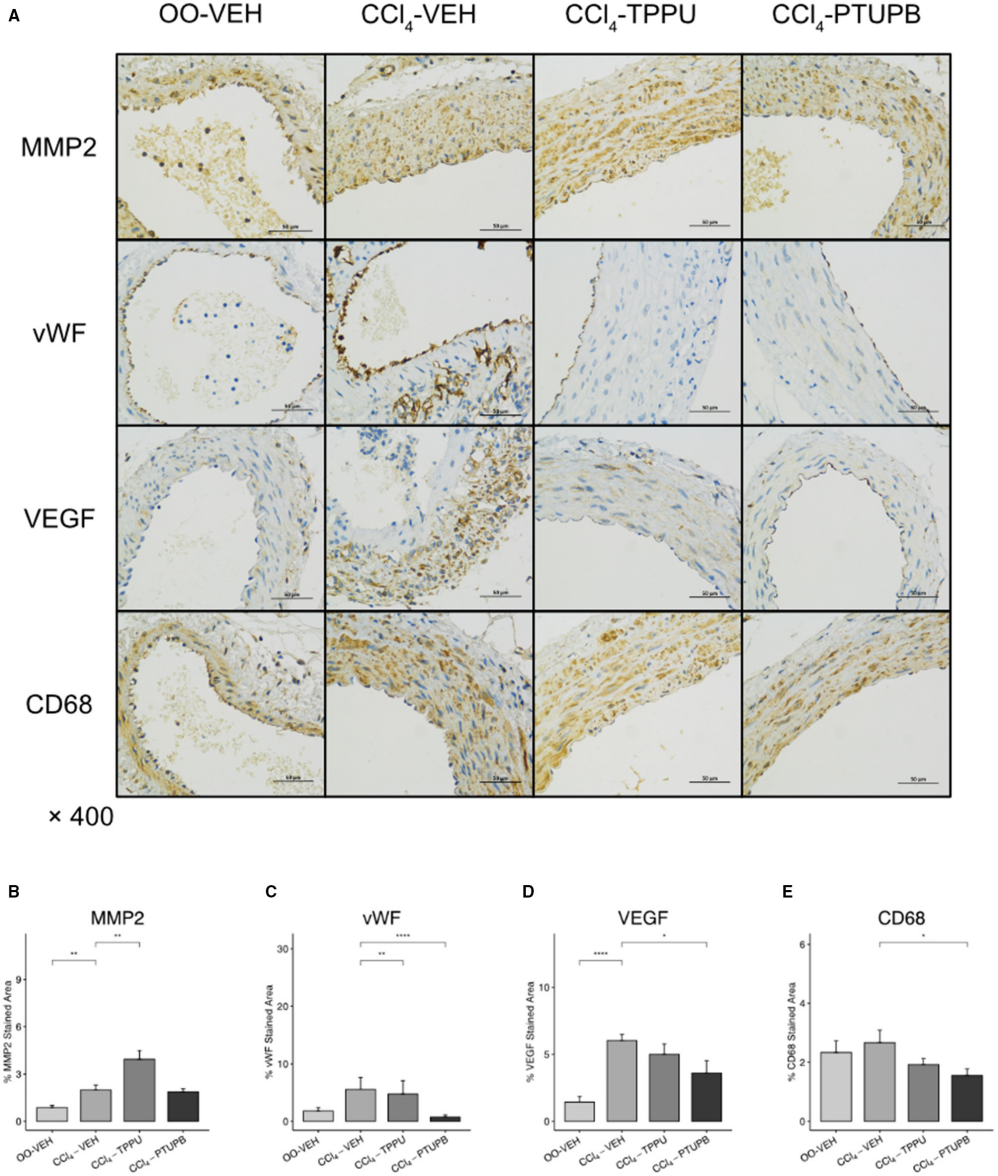
PTUPB inhibits vascular remodeling and inflammation in SMA. **(A)** IHC staining for MMP2, vWF, VEGF, and CD68 in SMA. Quantification of IHC staining of vWF **(C)**, VEGF **(D)**, and CD68 **(E)** decreased in CCl_4_-PTUPB group whereasMMP2 **(B)** showed no significant difference. (*p <0.05, **p <0.01, ****p <0.0001 vs. CCl_4_-VEH using Student's *t*-test, data are represented as mean with SD).

In addition, the expression of remodeling factors including MMP2 and VEGF in SMA also increased significantly in PHT group (Figures 6B,D). After PTUPB treatment, vWF, VEGF, and CD68 decreased significantly (Figures 6C–E). As a contrast, only vWF reduced significantly in TPPU group (Figure 6C).

### Molecular Mechanism of PTUPB in Alleviating Liver Fibrosis

**Figure 7:**
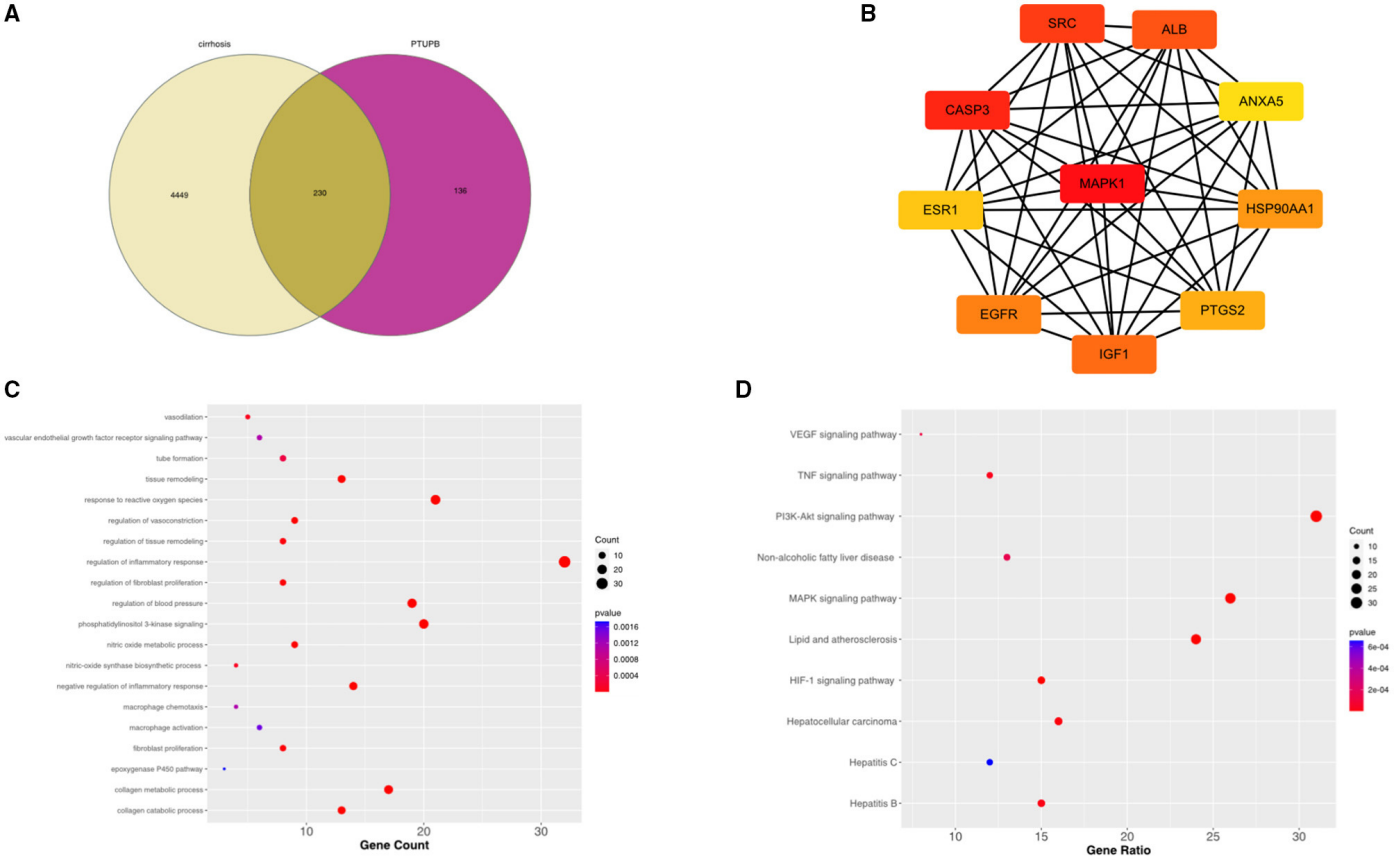
Intersected targets between PTUPB and liver fibrosis and enrichment analysis. **(A)** The intersection of liver cirrhosis (yellow) and PTUPB targets (red). **(B)** Top ten genes among the common target genes determined by degree centrality. **(C)** Top 20 biological processes from GO enrichment. The *x*-axis represented the gene counts, *y*-axis indicated the different GO terms, and the color of dots represented the *p*-values. **(D)** Top 10 enriched KEGG signaling pathways. The *x*-axis represented the gene ratio, which indicated the number of genes enriched in one pathway compared with the total genes, *y*-axis indicated the different terms, and the color of dots represented the *p*-values.

A total of 4,679 genes related to liver fibrosis were predicted through the GeneCards database. Based on the pharmacophore model and the principle of structural similarity, a total of 366 PTUPB-related target genes were collected through the SwissTargetPrediction and the PharmMapper database. After intersecting and merging cirrhosis-related genes and PTUPB predicted targets, 230 overlapping targets were obtained as candidate genes, which were shown in the Venn diagram (Figure 7A). To clarify the interactions among common targets between liver fibrosis and PTUPB, a PPI network was constructed by String database and CytoHubba plugin of Cytoscape. Using MCC method, the hub genes with the highest DC values were identified based on three topological parameters (degree, betweenness, and closeness centrality). The top 10 hub genes included are MAPK1, CASP3, SRC, ALB, IGF1, EGFR, HSP90AA1, PTGS2, ESR1, and ANXA5 (Figure 7B).

Subsequently, enrichment analysis was conducted on common target genes. In GO analysis, the common targets mainly involved in regulation of inflammatory response, phosphatidylinositol 3-kinase signaling, response to reactive oxygen species, regulation of vasoconstriction, regulation of blood pressure, vasodilation, negative regulation of inflammatory response, tissue remodeling, nitric oxide metabolic process, regulation of tissue remodeling, nitric oxide synthase biosynthetic process, tube formation, VEGF receptor signaling pathway, macrophage chemotaxis, macrophage activation, regulation of fibroblast proliferation, fibroblast proliferation, collagen metabolic process, collagen catabolic process, and epoxygenase P450 pathway (Figure 7C).

Kyoto Encyclopedia of Genes and Genomes results suggested that the enrichment pathways included VEGF signaling pathway, tumor necrosis factor (TNF) signaling pathway, non-alcoholic fatty liver disease (NAFLD), lipid and atherosclerosis, hypoxia inducible factor (HIF)-1 signaling pathway, hepatitis B, drug metabolism—CYP450, chemokine signaling pathway, apoptosis, and ARA metabolism (Figure 7D).

**Figure 8:**
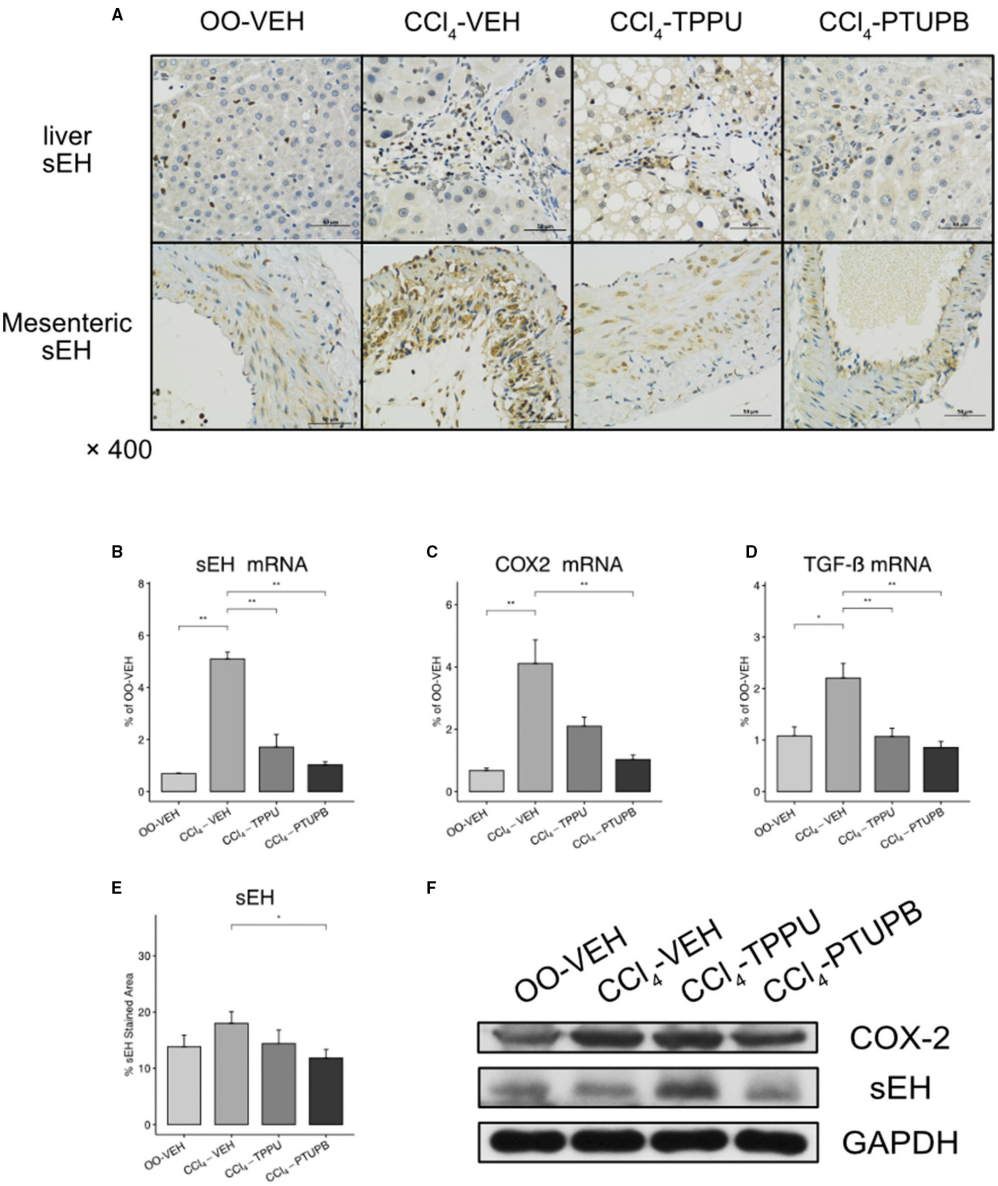
PTUPB inhibits the expression of sEH, COX-2, and TGF-β. **(A)** IHC staining for sEH in liver and SMA. The gene expression of sEH **(B)**, COX-2 **(C)**, and TGF-β **(D)** decreased significantly in CCl_4_-PTUPB group. **(E)** Quantification of IHC staining of sEH decreased in CCl_4_-PTUPB group. **(F)** Western blots of hepatic sEH and COX-2 expression (*p <0.05, **p <0.01 vs. CCl_4_-VEH using Student's *t*-test, data are represented as mean with SD).

As a dual COX-2/sEH inhibitor, we evaluated the inhibitory effect of PTUPB on COX-2/sEH pathways and proinflammatory cytokine TGF-β. In PHT rats, the hepatic expression of sEH, COX-2, and TGF-β increased significantly (Figures 8B–D). Besides, sEH staining in SMA also increased in PHT rats (A). Comparable results were found in western blot analysis (Figure 8F). After treatment with PTUPB, the expression of sEH, COX-2, and TGF-β in the liver decreased significantly (Figures 8B,C). Similar results were also seen in immunohistochemical analysis and western blot (Figures 8A,E,F). However, in the TPPU treatment group, only sEH and TGF-β decreased significantly, whereas COX-2 remained unchanged (Figures 8B–D,F).

## Discussion

As a dual sEH/COX-2 inhibitor, the beneficial effect of PTUPB in liver fibrosis and PHT was examined for the first time in our study. Several pharmacological effects of PTUPB were validated including antifibrosis, PP-lowering effect, antiinflammation, antiangiogenesis, sinusoidal vasodilation, and ameliorating vascular remodeling in sinusoids and SMA. The enrichment analysis indicated that PTUPB engaged in multiple biological functions related to liver fibrosis, including vasoconstriction, nitric oxide metabolic process, angiogenesis, blood pressure, tissue remodeling, immune inflammation, macrophage activation, fibroblast proliferation, and also collagen metabolism and CYP450 enzyme. The corresponding signaling pathways included VEGF, TNF, HIF, NAFLD, CYP450, hepatitis, etc. The inhibitory effects of PTUPB on sEH, COX-2, and TGF-β were also observed in our study. In summary, PTUPB has a substantial impact on liver fibrosis and PHT by suppressing inflammation, fibrosis, angiogenesis, and vascular remodeling.

In the treatment of cirrhosis, it is important to control tissue inflammation, fibrosis, and also vascular remodeling. As a critical component of the healing response to liver injury, inflammation is closely associated with the development of liver fibrosis. In this process, macrophage activation stimulates fibrosis by secreting a variety of proinflammatory cytokines such as IL-6 (21), which could further damage hepatocytes and exacerbate liver fibrosis (22). Our findings confirmed that IL-6 production, macrophage infiltration, and the number of bile ducts increased in liver fibrosis. Additionally, all those inflammation responses were alleviated effectively by PTUPB therapy.

The progression of liver fibrosis and inflammation causes intrahepatic resistance, hyperdynamic circulation, which leads to PHT eventually. In this process, the impact on hemodynamic parameters is divided into intra- and extrahepatic aspects. Similar to previous studies (23), multiple intrahepatic pathological alterations were observed in PHT including angiogenesis, vascular remodeling, and sinusoidal dysfunction. Our research revealed that PTUPB had a positive effect on hemodynamic parameters both structurally and functionally. Following PTUPB therapy, angiogenic and sinusoidal remodeling factors were found to be downregulated which include VEGF, vWF, CD31, and MMP2, whereas regulators of vascular tone such as GCH1 and eNOS were shown to be increased. According to previous study, VEGF, vWF, and MMP2 are major regulators of angiogenesis and vascular remodeling (24–26). CD31 is widely used to assess proliferation of endothelial cells and angiogenesis (27). GCH1 is a pivotal enzyme in the synthesis of eNOS. Both GCH1 and eNOS are important regulators of vascular tone (28).

Along with intrahepatic vascular in PHT, extrahepatic vascular undergoes structural alterations as well, including collateral angiogenesis, arterial vessel wall remodeling, etc. (2). A previous research indicated that cirrhotic PHT rats had a decrease in the wall thickness and total wall area of abdominal aorta (29), which was verified in SMA in our study. Our results showed that PTUPB treatment enhanced the thickness of the SMA wall, improved vascular remodeling, and decreased inflammatory markers including vWF, VEGF, and CD68. Furthermore, PTUPB decreased PPs in PHT rats as well. In conclusion, PTUPB had a favorable regulating effect on both intrahepatic and extrahepatic angiogenesis and vascular remodeling in cirrhotic PHT, which may explain its PP-lowering effect.

As previously stated, the ARA pathway plays a critical role in liver fibrosis, which was formerly characterized by two distinct pathways: COX-2 and sEH. COX-2 inhibitors such as SC-236 and meloxicam were shown to attenuate the development of liver fibrosis through cell apoptosis and TGF-β1 pathway, respectively (30, 31). However, Harris et al. (11) investigated the COX-2-selective inhibitor Celebrex in liver fibrosis and found no meaningful effect. Considering the mice model used in this study, COX-2 may have varying impacts on different stages of cirrhosis. Besides the COX-2, our group also found that sEH inhibition by t-TUCB increased eNOS levels, reduced inflammation, and alleviated cirrhotic PHT (32, 33). Furthermore, sEH-related pathways are involved in endothelial function, hypertension, and oxidative stress (6, 34, 35). As a key profibrotic factor, TGF-β is also engaged in sEH and COX-2 pathways (31, 36). This is consistent with our data, in which COX-2, she, and TGF-β were upregulated in liver fibrosis and decreased after the PTUPB treatment.

As a dual sEH/COX-2 inhibitor, PTUPB has been widely investigated in a variety of illnesses. Zhang et al. (9) discovered that PTUPB may decrease collagen deposition and ameliorate bleomycin-induced lung fibrosis through cellular senescence. Hye et al. (10) found that PTUPB can effectively alleviate renal injury, inflammation, and fibrosis in kidney injury models. In chronic liver disease, Sun et al. (37) found that PTUPB significantly reduced liver fibrotic deposition and inflammation in NAFLD mice induced by high-fat diet. These protective effects are mainly mediated through lipid metabolism, NLRP3 inflammasome, and steatosis. Furthermore, Harris et al. (11) revealed that PTUPB had a certain alleviating impact on liver fibrosis and inflammation in liver injury within CCl_4_ injection for 5 weeks. However, the effect of PTUPB on advanced liver cirrhosis, intra- or extrahepatic angiogenesis, and vascular remodeling, and also PHT, still remains unknown. Here, our study confirmed its therapeutic effect using cirrhotic rat PHT model through CCl4 injection for 16 weeks.

The limitations of the study stemmed mostly from its phenotypic verification rather than in-depth research of its molecular mechanism. In future, further experiments *in vitro* will be required to establish PTUPB's cellular targets and validate the pathways. In terms of hemodynamics, we assessed the blood pressure but were unable to measure the hepatic blood flow due to equipment and experimental technical constraints. Additionally, CCl_4_-induced liver cirrhosis is an artificial model that may be challenging to adapt to human disease prediction.

## Conclusion

In conclusion, our findings showed that PTUPB had a significant protective effect on liver fibrosis and PHT by inhibiting hepatic fibrotic deposition and inflammation, suppressing angiogenesis and vascular remodeling in sinusoids and SMA, and inducing sinusoidal vasodilation. The mechanism may be mediated *via* the downregulation of the sEH/COX-2/TGF-β. Thus, PTUPB represents a promising approach in the treatment of cirrhosis-related PHT.

## Data Availability Statement

The raw data supporting the conclusions of this article will be made available by the authors, without undue reservation.

## Ethics Statement

The animal study was reviewed and approved by Ethical Committee of Shanghai Ninth People's Hospital, Shanghai Jiao Tong University School of Medicine.

## Author Contributions

ZZ and CZ were involved in the plan of program and drafted the manuscript. JL and LZ participated in data collection and analysis. HL and XQ performed the experiment. BH, HH, and XL provided reagents or materials or analysis tools. SH and BH designed and synthesized PTUPB. YB, ZZ, and ML participated in drafting or revising the work. All authors have given the final approval of the version to be published and accountable for all aspects of the manuscript.

## Funding

This study is partially supported by NIH-NIEHS (RIVER Award) R35 ES030443-01, NIH-NIEHS (Superfund Award) P42 ES004699 (both to BH) and National Natural Science Fund of China (Project No. 82100639) (to LZ), (Project No. 81970526, 81770599) (to ML), (Project No. 81900550) (to HL).

## Conflict of Interest

The authors declare that the research was conducted in the absence of any commercial or financial relationships that could be construed as a potential conflict of interest.

## Publisher's Note

All claims expressed in this article are solely those of the authors and do not necessarily represent those of their affiliated organizations, or those of the publisher, the editors and the reviewers. Any product that may be evaluated in this article, or claim that may be made by its manufacturer, is not guaranteed or endorsed by the publisher.

## Abbreviations

ALT: alanine transaminase
ANGPT1: angiopoietin 1
ARA: arachidonic acid
AST: aspartate aminotransferase
CCl4: carbon tetrachloride
COL1A1: collagen type I alpha 1 chain
COX-2: cyclooxygenase-2
CYP450: cytochrome P450
CK-19: cytokeratin 19
DBIL: direct bilirubin
eNOS: endothelial nitric oxide synthase
ELISA: enzyme-linked immunosorbent assay
EPHX2: epoxide hydrolase 2
EETs: epoxyeicosatrienoic acids
ECM: extracellular matrix
GCH1: GTP-cyclohydrolase 1
HR: heart rate
IL-6: interleukin-6
LOX: lipoxygenase
MMP-9: matrix metalloproteinase-9
MMP-2: matrix metalloproteinase-2
MAP: mean arterial pressure
PBS: phosphate-buffered saline
PVDF: polyvinylidene difluoride
PHT: portal hypertension
PP: portal pressure
PPI: protein–protein interaction
she: soluble epoxide hydrolase
SD: Sprague Dawley
SMA: superior mesenteric arteries
TBIL: total bilirubin
TGF-β: transforming growth factor-β
VEGF: vascular endothelial growth factor
vWF: von Willebrand factor
GGT: γ-glutamyl transferase.
